# Transcriptome analysis of *Echinococcus granulosus *sensu stricto protoscoleces reveals differences in immune modulation gene expression between cysts found in cattle and sheep

**DOI:** 10.1186/s13567-022-01022-3

**Published:** 2022-01-28

**Authors:** Ismael Pereira, Christian Hidalgo, Caroll Stoore, María Soledad Baquedano, Carolina Cabezas, Macarena Bastías, Aníbal Riveros, Claudio Meneses, Martín Cancela, Henrique Bunselmeyer Ferreira, Leonardo Sáenz, Rodolfo Paredes

**Affiliations:** 1 Laboratorio de Medicina Veterinaria, Escuela de Medicina Veterinaria, Facultad de Ciencias de la Vida, Universidad Andres Bello, Santiago, Chile; 2 Programa de Doctorado en Ciencias Silvoagropecuarias y Veterinarias, Campus Sur Universidad de Chile, Santa Rosa 11315, La Pintana, 8820808 Santiago, Chile; 3 Instituto de Ciencias Agroalimentarias, Animales y Ambientales (ICA3), Universidad de O’Higgins, San Fernando, Chile; 4 Centro de Biotecnología Vegetal, Facultad de Ciencias de la Vida, Universidad Andres Bello, Santiago, Chile; 5 Laboratório de Genômica Estrutural e Funcional, Centro de Biotecnologia, Universidade Federal do Rio Grande do Sul, Porto Alegre, 91501-970 Brazil; 6 Laboratorio de Vacunas Veterinarias, Facultad de Ciencias Veterinarias y Pecuarias, Universidad de Chile, Santiago, Chile

**Keywords:** Cystic echinococcosis, RNAseq, transcriptomics, protoscolex, cattle, sheep, immune modulation

## Abstract

Cystic Echinococcosis (CE), a zoonotic parasitic disease, is caused by the cestode *Echinococcus granulosus *sensu lato. CE inflicts severe damage in cattle, sheep, and human hosts worldwide. Fertile CE cysts are characterized by the presence of viable protoscoleces. These parasite forms are studied with minimal contamination with host molecules. Hosts, cattle and sheep, show differences in their CE cyst fertility. The effect of the host in protoscolex transcriptome is not known. We genotyped and performed transcriptomic analysis on sheep protoscoleces obtained from liver and lung CE cysts. The transcriptomic data of *Echinococcus granulosus *sensu stricto protoscoleces from 6 lung CE cysts and 6 liver CE cysts were Collected. For host comparison analysis, 4 raw data files belonging to *Echinococcus granulosus *sensu stricto protoscoleces from cattle liver CE cysts were obtained from the NCBI SRA database. Principal component and differential expression analysis did not reveal any statistical differences between protoscoleces obtained from liver or lung cysts, either within the same sheep or different sheep hosts. Conversely, there are significant differences between cattle and sheep protoscolex samples. We found differential expression of immune-related genes. In cattle, 7 genes were upregulated in protoscoleces from liver cysts. In sheep, 3 genes were upregulated in protoscoleces from liver and lung CE cysts. Noteworthy, are the differential expression of antigen B, tegument antigen, and arginase-2 in samples obtained from sheep CE cysts, and basigin in samples from cattle CE cysts. These findings suggest that the host species is an important factor involved in the differential expression of immune related genes, which in turn is possibly related to the fertility of *Echinococcus granulosus *sensu stricto cysts.

**Supplementary Information:**

The online version contains supplementary material available at 10.1186/s13567-022-01022-3.

## Introduction

Cystic echinococcosis (CE), caused by infection with the metacestode stage (CE cyst) of the flatworm *Echinococcus granulosus *sensu lato (s.l.), is a major zoonotic disease with worldwide distribution. This Neglected Tropical Disease (NTD) is included by the World Health Organization (WHO) as a part of a neglected zoonosis subgroup with strategic plans for control since 2008 [1]. The life cycle of the parasite uses the canids as definitive hosts and herbivores as intermediate hosts where the metacestode is formed. CE cysts are a fluid filled structure, comprised of 3 layers: adventitial layer (formed by host-parasite reaction), laminated layer and the inner layer called germinal layer, this last layer and its cellular component differentiate to the infective stage, the protoscolex (PSC) [2]. In canids, infection is acquired by consuming PSC from raw CE cysts from livestock offal, whereas intermediate hosts acquire the infection by consuming eggs shed from canids. The low host specificity of this parasite is reflected in the large list of intermediate hosts, even including abdominal cysts in domestics cats [3].

For reasons not fully understood, CE cysts in different species of intermediate hosts and anatomical location, show different PSC production capabilities. In some species, such as sheep, CE cysts are formed with a high capacity to produce PSC (fertile CE cysts), whereas in other hosts, such as cattle, CE cyst usually do not have viable PSC, known as non-fertile CE cysts [4, 5]. Although different species of *E. granulosus* s.l. are associated with a particular intermediate host, such as *E. granulosus *sensu stricto (s.s.) with sheep and *E. ortleppi* with cattle [6], both fertile and non-fertile CE cysts are generated in cattle and sheep with *E. granulosus* s.s. [7]*.* and *E. ortleppi* [8]. Another factor involved in CE cyst fertility is the host immune response in the adventitial layer. Granulomatous reactions are associated with low PSC viability and non-fertile CE cysts, and fibrotic resolutions are associated with fertile CE cysts [9, 10].

Transcriptomic analysis and next generation sequencing are useful tools to understand many cellular and physiological processes in platyhelminths, such as immune modulation response. In *Fasciola gigantica*, it is useful in identifying differentially expressed genes between juvenile and adult stages, where the expression of transcripts by juvenile parasites that trigger the TGF-β pathway in host cells are associated with evading protective immunity [11]. This finding was confirmed in transcriptomic studies from infected mammalian host cells [12, 13]. In cestodes, immune modulation genes were detected in adult and metacestode stages of *Taenia pisiformis*, with cathepsins identified as a key parasite molecule involved in immune modulation strategies of the adult form [14, 15]. Many works published in transcriptomic analysis of *E. granulosus *s.l. protoscolex, focus on the differential expression of excretory-secretory (ES) products [16], alternative splicing [17] and genes involved in the bi-directional transformation to either adult or cystic forms [18–20]. However, these analyses did not explore the response of the parasite to different microenvironments (lung or liver tissue) or the differential gene expression related to immune modulation by the parasite itself. Oudni-M’rad et al. [21] reported that *E. granulosus* s.s. protoscoleces from lung and liver CE cysts are genetically different populations and both, the host species and the localization (lung or liver) were the most important factors for genetically differentiating PSC.

The publication of the genome and transcriptome of *E. granulosus* s.l. provides possibilities to explore the responses of the parasite to the host in a transcriptomic way. Since PSC viability is the defining criteria for CE cyst fertility [22] and it is associated with the host species and immune response [9], we studied the different gene expression patterns with a focus on the immune modulation genes of *E. granulosus* s.s. protoscoleces from sheep and cattle CE cysts.

## Materials and methods

### Sample collection and viability

Twelve fresh CE cysts were collected from sheep (6 from the liver and 6 from the lungs) at local abattoirs. All CE cysts were inspected visually, measured, and each one was considered as an independent sample. Only cysts between 3 and 10 cm in diameter were included. PSC obtained from lung CE cysts were labeled Lu01–Lu06 and PSC obtained from liver CE cysts samples were labeled Li01–Li06. To minimize variability, samples Lu01, Lu02, Lu03, Li01, Li02, and Li03 were obtained from the same animal (three lung CE cysts and three liver CE cysts), and the remaining samples were obtained from different sheep. After evaluating PSC viability with the trypan blue exclusion test, only PSC with  >90% viability were selected. From fertile CE cysts, PSC were washed in a PBS pH 7.4 and then transferred to RNAlater^©^ solution and frozen at −80 °C for storage until analysis. Two groups comprised of 6 biological replicates were made: 6 samples of PSC from sheep liver CE cysts and 6 samples of PSC from sheep lung CE cysts.

### DNA isolation and genotype identification

The DNA isolation was performed with WIZARD Plus SV Genomic Purification Systems kit (PROMEGA). Genotype identification was performed with a PCR using 30–100 ng of DNA, and 0.5 U DNA Taq Pol, 1X Buffer Taq DNA Pol (20 mM Tris–HCl, pH 8.4, 50 mM KCl), 0.04 mM mix dNTP, 1.5 mM MgCl_2_, and 20 pmol of each primer (5′-TTA CTG CTA ATA ATT TTG TGT CAT-3′ forward and 5′-GCA TGA TGC AAA AGG CAA ATA AAC-3′ reverse), targeting the full length of the cytochrome C oxidase subunit 1 (cox1, 1609 bp) in a final volume of 25 µL. After amplification, PCR products were purified and sequenced by Sanger sequencing. These samples were aligned to the Eg01 haplotype as the reference sequence (Accession No. JQ250806) [23], with manual verification of peaked data when differences were found. Only confirmed *E. granulosus *s.s. samples were used.

### RNA isolation, cDNA library preparation and Seq summary

Total RNA was extracted with Rneasy^®^ mini kit and quantified by fluorometry (Qubit 2.0, Invitrogen). The inclusion criteria were the following: minimum high purity (A260/A280  > 1.8), 1 µg of total RNA, and an electropherogram pattern compatible to those reported elsewhere [24]. Libraries were built using Illumina TruSeq^®^ Stranded mRNA Library Prep Kit, following the manufacturer’s protocol. These libraries were sequenced in paired-end (2 × 100 bp) on Illumina HiSeq4000 platform. In the raw samples there was a mean of 6.5615 Gb in total bases and a read count of 64.965 M. In relation to raw data quality 98.14% of the samples fitted in the Q20 Phred score and 94.74% fitted in the Q30 Phred score. With the raw reads, we performed several steps before performing a differential expression analysis (DEA) with DeSEQ2 [25]. Briefly, trimming was done with TrimGalore, then Quality control with FastQC was made [26]. The alignment was made with STAR software and using the *E. granulosus* genome version GCA_000524195.1 ASM52419v1 as a reference, getting a mean of 88.72% of uniquely mapped reads, 2.07% multiple loci mapped reads and a total of 91.01% of mapped reads. The Matrix Count was generated with feature Counts. Finally the gProfiler web server [27] was used for gene ontology (GO) analysis (Additional files 1, 2).

### Short read archive (SRA) cattle PSC data

For cattle CE cyst samples, 4 raw data files belonging to *E. granulosus* s.s. PSC from cattle liver CE cysts were obtained from the NCBI SRA database under the access number PRJNA432155. Samples were labeled B1-B4 and consisted of PSC washed with PBS (B1), treated with pepsin (B2), and cultured in biphasic medium for 12 h (B3) or 24 h (B4) [19].

### Bioinformatics analysis

RAW files were adapter trimmed with TrimmGalore to remove low quality sequences, primers and adapters and trimmed to a set length cutoff of 50 bp. Quality control was performed with FastQC, the alignment was made with STAR and the reference genome was GCA_000524195.1 ASM52419v1. After the sorting (with samtools) and getting the counts matrix with Rsubread/featureCounts, a DEA was performed with DESeq2. A gene was considered differentially expressed with FDR  < 0.05 and a log_2_FoldChange  > 1.5.

### Quantitative real-time PCR (qRT-PCR)

Reverse transcription was done with ImProm-II Promega reverse transcription kit, following the manufacturer’s protocol. For qRT-PCR, 250 ng of cDNA were used with primers for selected genes (Additional file 7). Relative quantification was performed using the PowerUp Sybr Master Mix (Thermofisher) and the StepOne detection system (Applied Biosystems) Reaction mix was done as follows: 10μL of PowerUp Sybr Master Mix, 0.5/1 μL forward primer, 0.5/1 μL reverse primer (according to primer efficiency, for 250 μM, 0.5 μL were used and for 500 μM, 1 μL was used), and nuclease-free water to complete 20 μL. Using the following program: 95 °C for 10 min, 40 cycles of 95 °C for 15 s, 60 °C for 30 s and 72 °C for 20 s, following a denaturation step of 95 °C to check for amplification specificity. TBP gene (EGR_02554) was used as the reference gene [28].

## Results

### Genotype and haplotype identification

**Figure 1:**
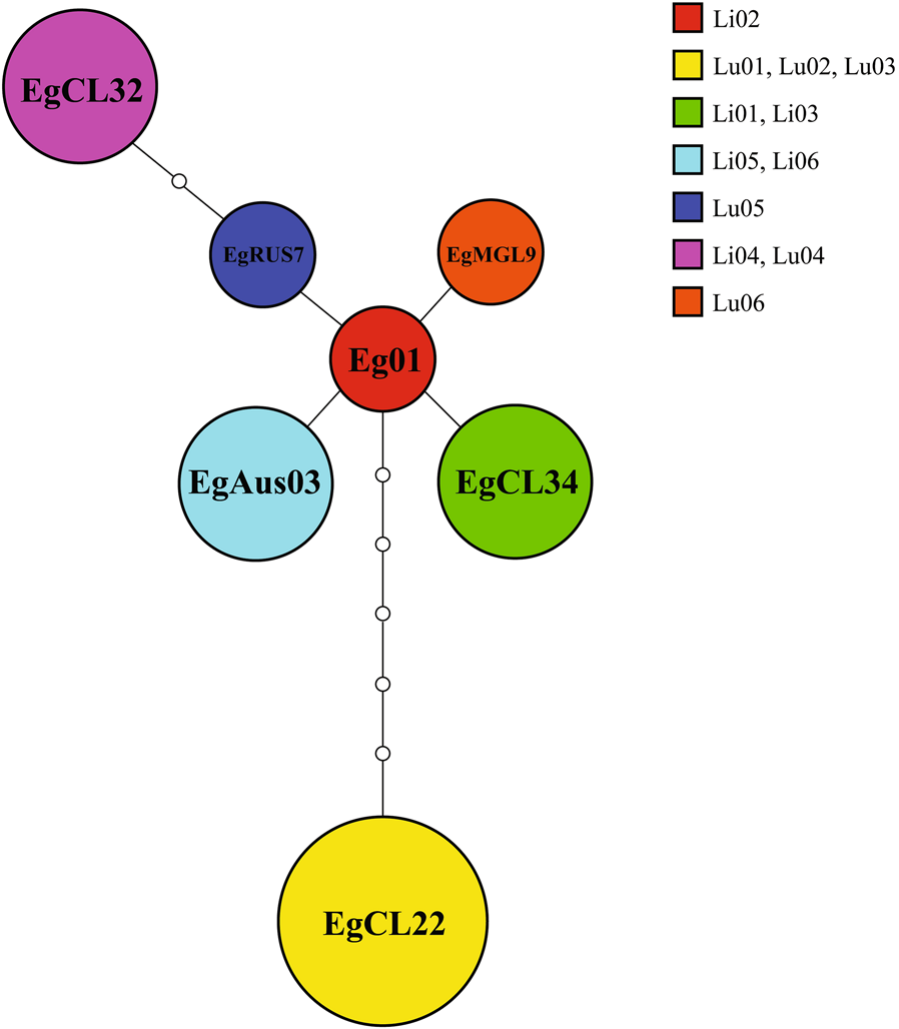
**Haplotype network of *Echinococcus granulosus *sensu stricto protoscoleces obtained from sheep liver and lungs cysts.** The network is built with the founder haplotype “Eg01” in the center, and each circle represents a difference in 1 nucleotide. The size of the circle is proportional to the number of samples of the same haplotype. Li01-06 = Protoscoleces from liver cysts. Lu01–06  =  Protoscoleces from lung cysts. Samples Li01–03 and Lu01–03 were obtained from the same animal.

After cox1 gene sequencing and alignment, 12 samples were confirmed to belong to the genotype *E. granulosus* s.s. Sequences were compared with the cox1 1609 bp sequences available in GenBank for cox1 haplotype identification. The PSC samples obtained from sheep liver CE cysts, Li01 and Li03 belong to haplotype EgCL34 (Accession No. MZ645038), Li02 to Eg01 (Accession No. JQ250806), Li04 to EgCL32 (Accession No. MK399402.1), Li05 and Li06 to EgAus03 (Accession No. KT968704.1). The PSC samples obtained from sheep lung CE cysts, Lu01-03 belong to haplotype EgCL22 (Accession No. MK139300.1), Lu04 to EgCL32 (Accession No. MK399402.1), Lu05 to EgRUS7 (Accession No. AB777904.1) and Lu06 to EgMGL9 (Accession No. AB893250.1). The haplotype network is shown in Figure 1.

### Differential expression between PSC from sheep liver and lung CE cysts

**Figure 2:**
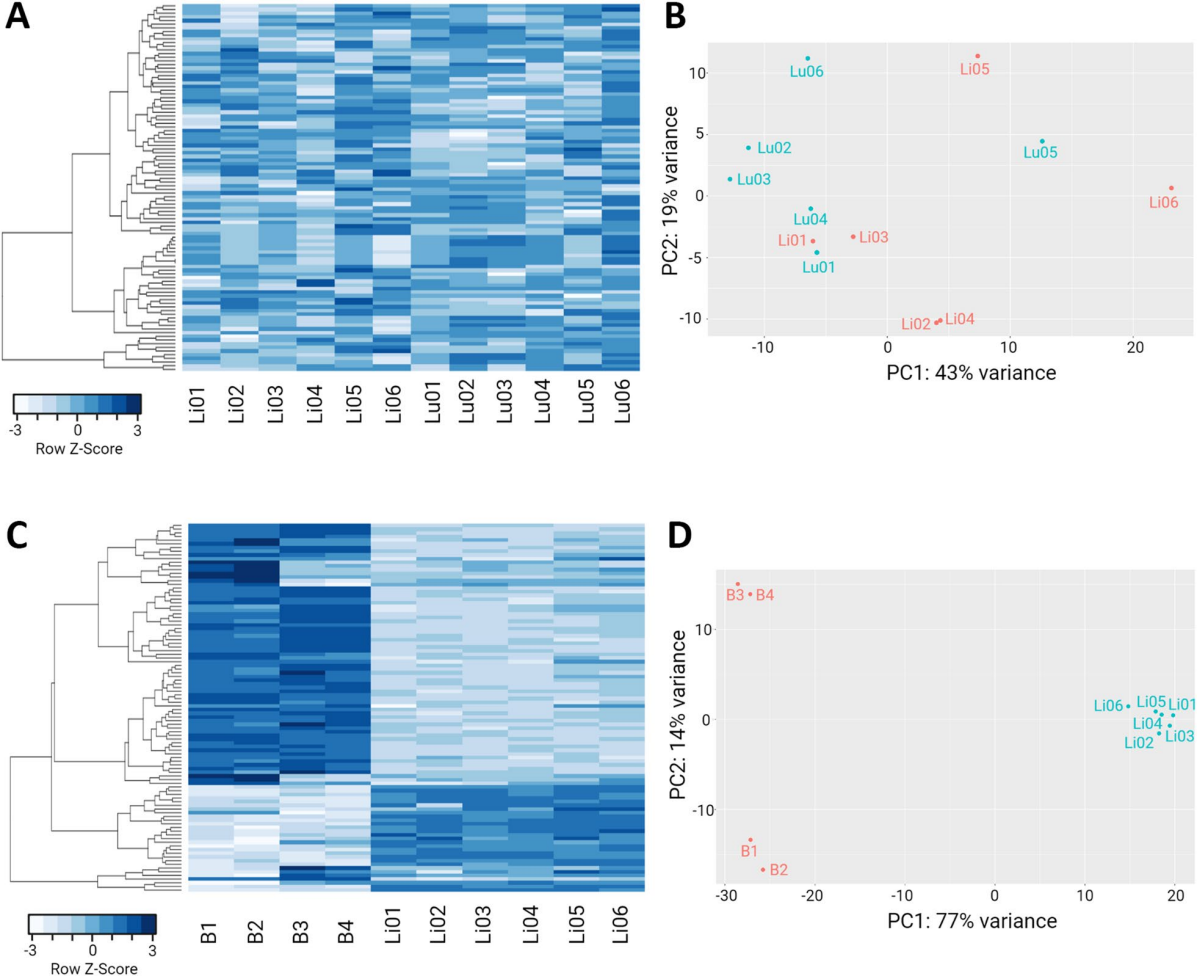
**Heatmap and principal component analysis (PCA) of expressed genes in protoscoleces from liver and lungs CE cysts from cattle and sheep. A** Heatmap of the top 100 most variable expressed genes in PSC from sheep liver and lung CE cysts. PSC from sheep liver CE cysts are labeled Li01–06 and PSC from sheep lung CE cysts labeled as Lu01–06. **B** PCA of PSC samples from sheep liver CE cysts v/s PSC samples from sheep lung CE cysts; The red dots represent Li01–06 samples and the turquoise dots Lu01–06 samples. **C** Heatmap of the top 100 most variable expressed genes between PSC samples from cattle and sheep liver CE cysts. Samples B1–4 are from cattle liver CE cysts PSC and Li01–06 from PSC samples from sheep liver CE cysts. **D** PCA of PSC samples from cattle liver CE cysts v/s PSC samples from sheep liver CE cysts; Red dots represent B1–4 samples and turquoise dots the Li01–06 samples. Samples Lu01, Lu02, Lu03, Li01, Li02, and Li03 were obtained from the same animal. CE  =  cystic echinococcosis.

To evaluate the heterogeneity among PSC from sheep liver and lung CE cysts, we performed a principal component analysis (PCA) and a heatmap with the top 100 genes most variably expressed among samples (Figures 2A, B). The results show a marked heterogeneity among samples, as the PCA did not exhibit any kind of clusters, even in samples from the same animal and cox 1 haplotype (Lu01, Lu02, and Lu03). Furthermore, the heatmap and its dendrogram, did not show any clear pattern of gene expression, demonstrating an important heterogeneity among groups or from samples from the same group.

**Figure 3:**
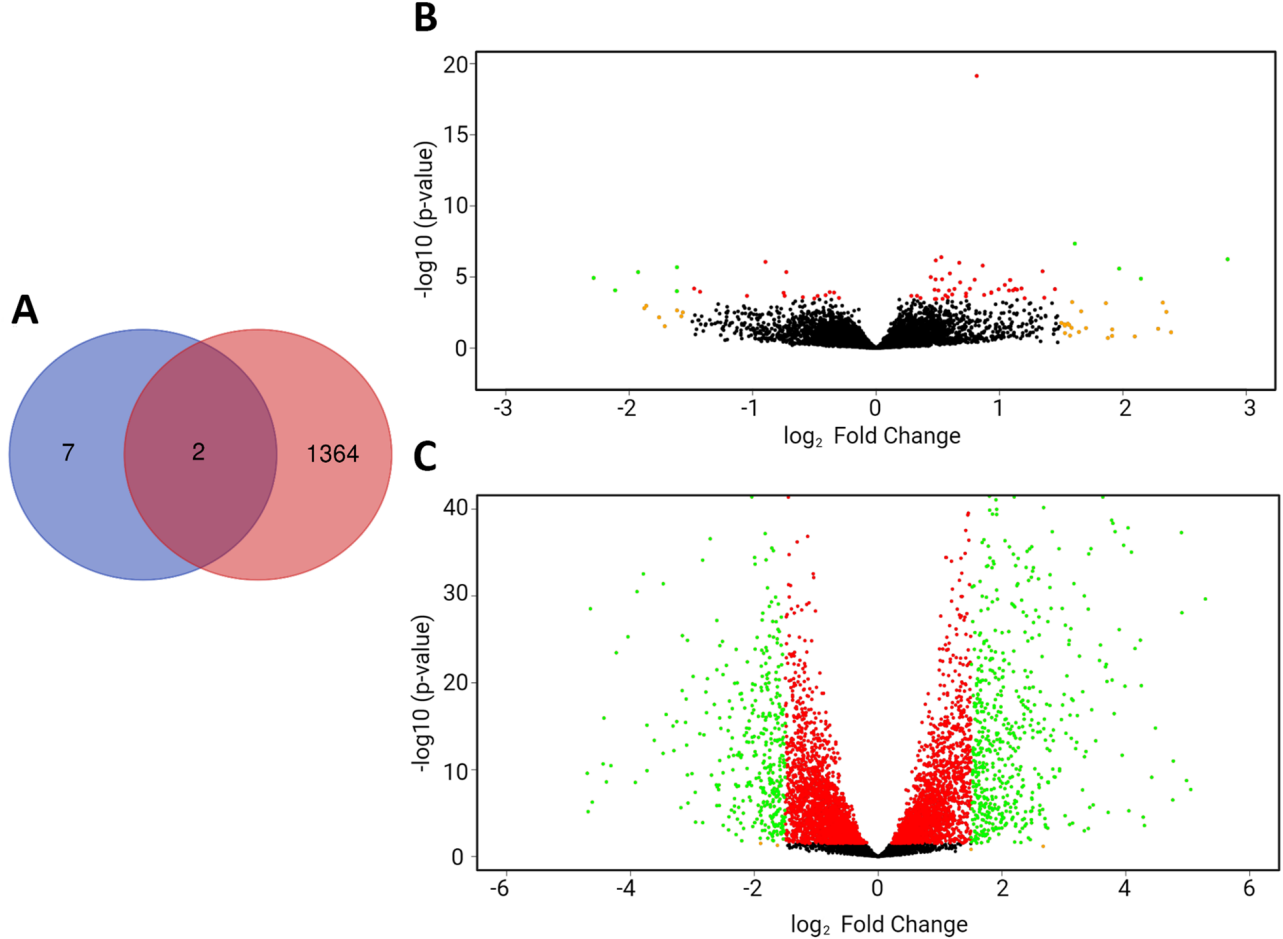
**Venn diagram and volcano plots of differentially expressed (DE) genes in protoscoleces from cattle and sheep liver and lung CE cysts. A** Venn diagram showing the number of DE genes for each group compared. The number of DE genes in PSC from sheep liver and lung CE cyst samples (9 genes) are in purple. The number of DE genes in PSC from cattle and sheep liver CE cyst samples (1366 genes) are in red. Two genes are shared between both comparisons. **B** Volcano plot of DE genes. Each dot is a gene. Negative values belong to PSC from sheep lung CE cyst samples, positive values belong to PSC from sheep liver CE cyst samples. Green dots: FDR  < 0.05 and log_2_FoldChange  > 1.5, Orange dots: FDR  > 0.05 and log_2_FoldChange  > 1.5, Red dots FDR  > 0.05 and log_2_FoldChange  < 1.5. Black dots do not fit any of these criteria. **C** Volcano plot of DE genes. Each dot is a gene. Negative values belong to PSC from sheep liver CE cyst samples, positive values belong to PSC from cattle liver CE cyst samples. Green dots: FDR  < 0.05 and log_2_FoldChange  > 1.5, Orange dots: FDR  > 0.05 and log_2_FoldChange  > 1.5, Red dots FDR  > 0.05 and log_2_FoldChange  < 1.5. Black dots do not fit any of these criteria.

In a detailed evaluation of the differentially expressed genes among these groups, four genes were upregulated in Li01-06 samples [EGR_10668, EGR_10232, EGR_10229 and EGR_03766 (all of them hypothetical proteins)] and five genes were upregulated in Lu01-06 samples [EGR_03387 (muscle M-line assembly protein unc-89)], EGR_03389 [subfamily M23B non-peptidase (M23 family), EGR_09714 (hypothetical protein), EGR_03390 (titin) and EGR_07198 (hypothetical protein EGR_07198)] with the criteria of FDR  < 0.05 and a log_2_FoldChange  > 1.5 (Figure 3). The rest of the differentially expressed genes are found in Additional file 3. Due to the almost null difference of the groups, an enrichment analysis was not performed.

### Differential expression between cattle and sheep PSC samples from liver CE cysts

We also performed a PCA and a heatmap with the top 100 genes most variably expressed among samples to evaluate heterogenicity. The results show a homogeneity of the samples per group revealing clustering of samples in the PCA and a similar pattern of gene expression in the heatmap of the top 100 genes most variably expressed among samples (Figures 2C, D).

We found 574 genes were upregulated in PSC from cattle CE cysts and 792 genes upregulated in PSC from sheep CE cysts, with FDR  < 0.05 and a log_2_FoldChange  > 1.5 (Figure 3). Two genes are shared among this and the previous comparison: EGR_03387 (Muscle M-line assembly protein unc-89) is upregulated in PSC from sheep lung CE cysts compared to PSC from sheep liver CE cysts EGR_03390 (Titin), is upregulated in PSC from sheep liver CE cysts compared to PSC from cattle liver CE cysts. The full list of differentially expressed genes is found in Additional file 4.

### Signaling pathways and GO terms in PSC obtained from sheep liver CE cysts and cattle liver CE cysts

**Figure 4:**
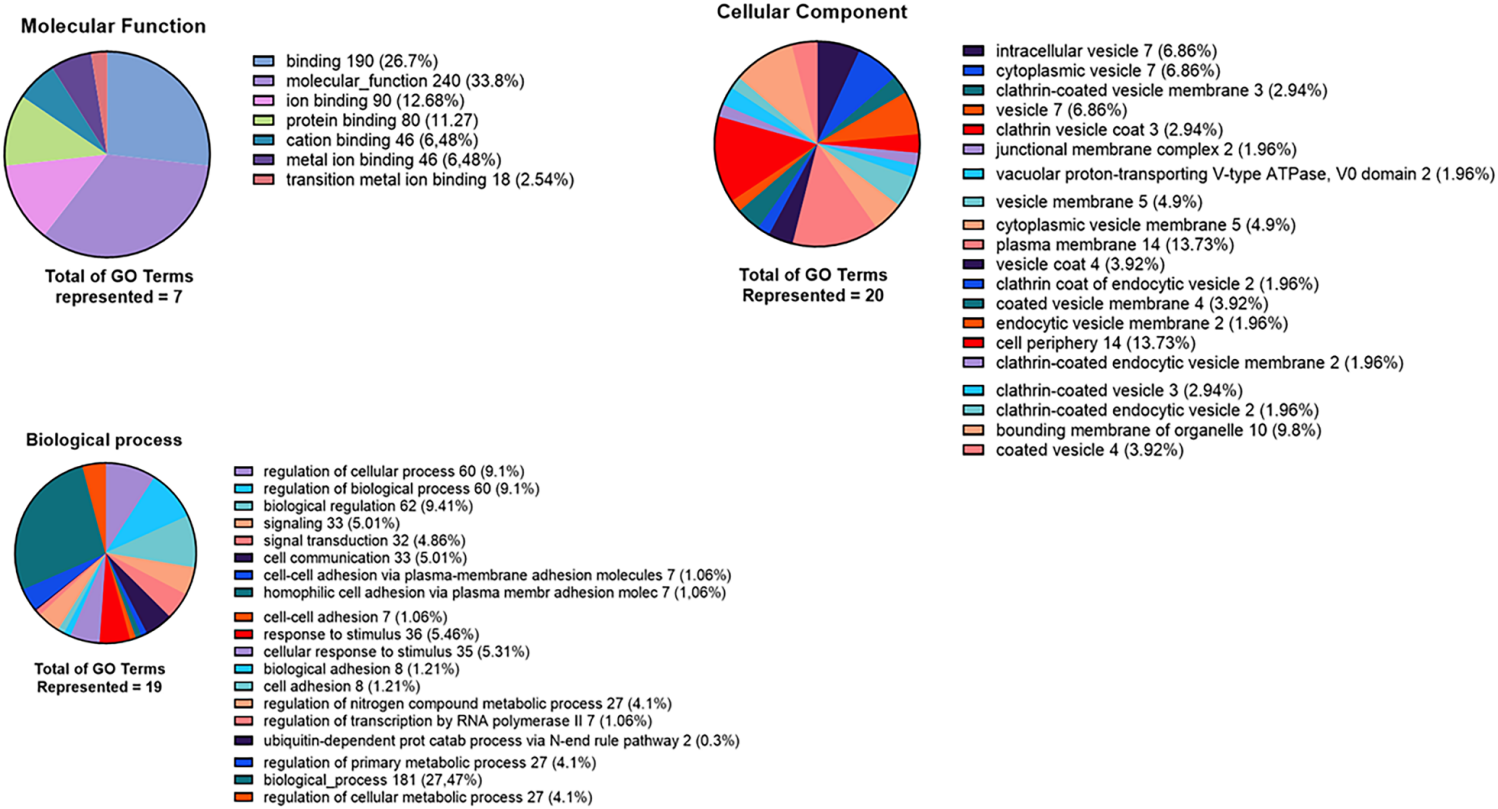
**Number and percentage by category (molecular function, biological process, and cellular component) GO terms in protoscoleces from sheep liver CE cysts.** GO terms from the comparison of PSC from sheep liver CE cysts v/s PSC from cattle liver CE cysts, with FDR 0.05 and without considering log_2_FoldChange cut-off. CE  =  cystic echinococcosis, PSC  =  protoscoleces.

**Figure 5:**
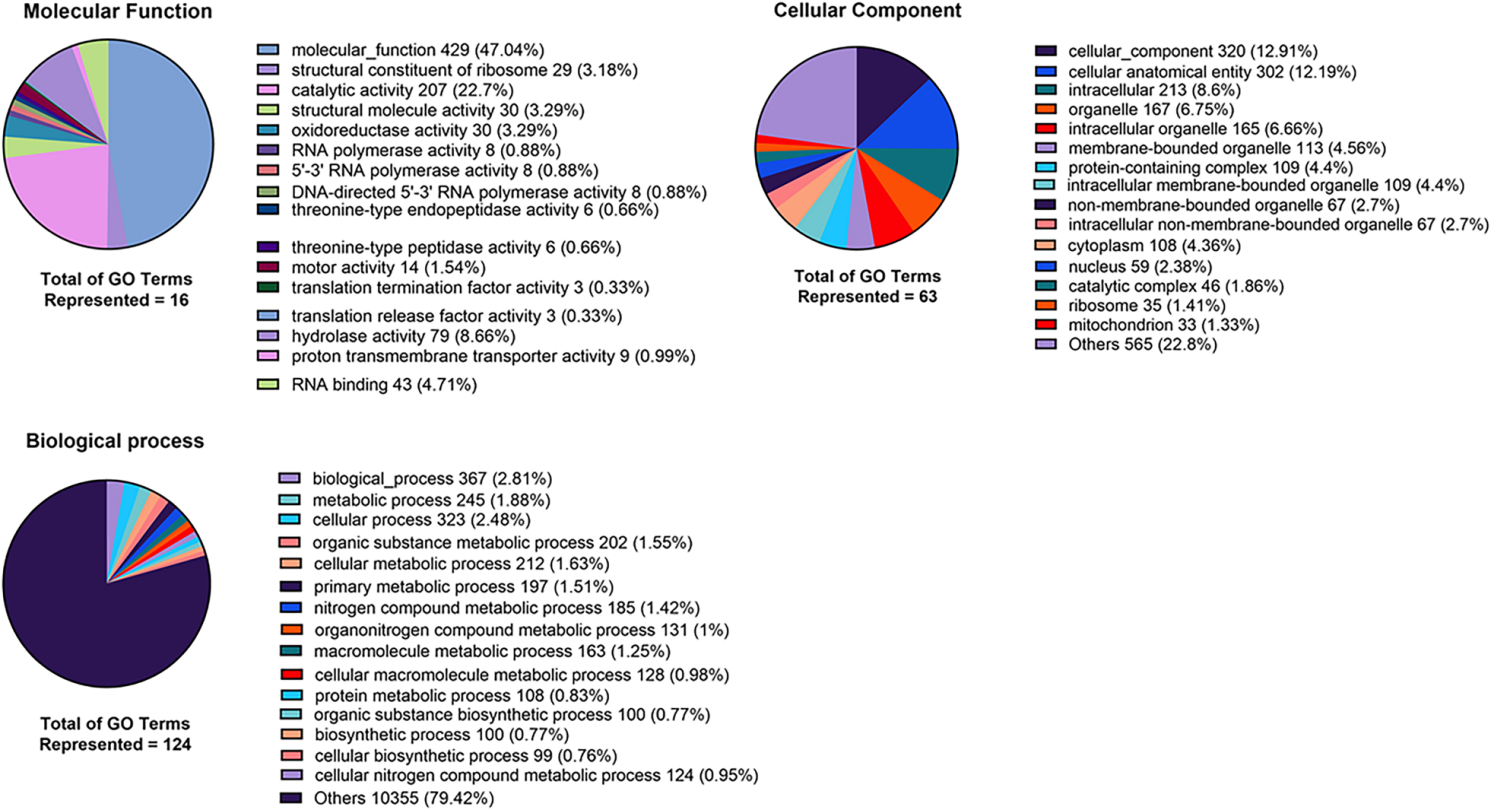
**Number and percentage by category (molecular function, biological process, and cellular component) GO terms in protoscoleces from cattle liver CE cysts.** GO terms from the comparison of PSC from cattle liver CE cysts v/s PSC from sheep liver CE cysts, with FDR 0.05 and without considering log_2_FoldChange cut-off. CE  =  cystic echinococcosis, PSC  =  protoscoleces.

We performed GO analysis for each group of upregulated genes, with a cut-off value of FDR  < 0.05 and log_2_FoldChange  > 0. For PSC sheep CE cysts, the top 3 GO terms per category were, in the Molecular function, “Transition metal ion binding” (GO:0046914) with 9% of the term size, “Metal ion binding” (GO:0046872) with 7% and “Cation binding” (GO:0043169) with 7%. In the Biological process category, we found “ubiquitin-dependent protein catabolic process via the N-end rule pathway” (GO:0071596) with 100%, “cell–cell adhesion via plasma-membrane adhesion molecules” (GO:0098742) with 18% and “homophilic cell adhesion via plasma membrane adhesion molecules” (GO:0007156) with 18%. In the cellular component category, we found “Junctional membrane complex” (GO:0030314) with 100%, “Vacuolar proton-transporting V-type ATPase V0 domain” (GO:0000220) with 100% and “Clathrin coat of endocytic vesicle” (GO:0030128) with 67%. In PSC from cattle CE cysts, the top 3 GO terms per category were, in the Molecular function: translation “Termination factor activity” (GO:0008079) with 75%, “Translation release factor activity” (GO:0003747) with 75% and “Threonine-type endopeptidase activity” (GO:0004298) with 40%. In the Biological process category, we found “Chaperone-mediated protein transport” (GO:0072321) with 100%, “Tetrahydrofolate biosynthetic process” (GO:0046654) and “Signal peptide processing” (GO:0006465), “Meiotic cell cycle process” (GO:1903046), “Meiotic nuclear division” (GO:0140013) with 75%. Finally, in the category Cellular component we found the following: “Signal peptidase complex” (GO:0005787) with 75%, “TRAPP complex” (GO:0030008) with 75% and “Proton-transporting ATP synthase complex, coupling factor F(o)” (GO:0045263) with 67%. A detail of all GO terms found and the number of genes per GO term may be seen in Figures 4,  5 and Additional file 5.

### Differential expression of immune modulation related genes between PSC obtained from cattle and sheep liver and lung CE cysts

After characterizing the DE of the two previous groups, we focused the search on immune modulation related genes, selecting 29 genes based on available DE genes with GO terms of immune modulation (Additional file 6).

Comparing PSC from sheep liver v/s lung CE cysts, we found that Antigen B (EGR_06805) was upregulated in PSC from sheep liver CE cysts, reaching the criteria of FDR  < 0.05 but not the log_2_FoldChange  > 1.5 criteria. In the comparison between PSC from cattle v/s sheep liver CE cysts we found 7 genes upregulated in PSC from cattle CE cysts, namely those coding for antigen B (EGR_09061 and EGR_06806), tegument antigen (EGR_08443), dolichyl-P-Man:Man(7)GlcNAc(2)-PP-dolichyl-alpha-1,6-mannosyltransferase (EGR_00930), dolichol-phosphate mannosyltransferase (EGR_01475), chitobiosyldiphosphodolichol beta-mannosyltransferase (EGR_04226), and arginase-2 (EGR_06681). The other 3 genes were upregulated in PSC from sheep CE cysts, namely those coding for basigin (EGR_08038), GPI mannosyltransferase (EGR_01290), and cathepsin L1 (EGR_02699). The results are summarized in Tables 1, 2.

**Table 1:** **Differentially expressed immune modulation genes between protoscoleces from sheep liver and lungs CE cyst samples**.

GeneID	Protein product	baseMean	log_2_FoldChange	lfcSE	Stat	*p* value	padj
EGR_09061	Antigen B	2101.83	10.12	0.97	10.40	2.4594E−25	3.47E−23
EGR_06806	Antigen B	5974.44	5.81	1.04	5.56	2.67E−08	3.01E−07
EGR_08443	Tegument antigen	1056.61	3.14	0.65	4.80	1.62E−06	1.21E−05
EGR_00930	Dolichyl-P-Man:Man(7)GlcNAc(2)-PP-dolichyl-alpha-1,6-mannosyltransferase	301.30	1.98	0.46	4.34	1.40E−05	8.32E−05
EGR_01475	Dolichol-phosphate mannosyltransferase	1898.75	1.39	0.25	5.51	3.61E−08	3.93E−07
EGR_04226	Chitobiosyldiphosphodolichol beta-mannosyltransferase	1860.16	1.18	0.20	5.76	8.24E−09	1.03E−07
EGR_06681	Arginase-2	43.33	5.79	0.57	10.08	7.04E−24	8.90E−22

FDR  < 0.05.

**Table 2:** **Differentially expressed immune modulation genes between protoscoleces from cattle and sheep liver CE cyst samples**.

GeneID	Protein product	baseMean	log_2_FoldChange	lfcSE	Stat	*p* value	padj
EGR_08038	Basigin	4219.37	−0.63	0.23	−2.74	6.22E−03	1.69E−02
EGR_01290	GPI mannosyltransferase	1526.55	−0.94	0.20	−4.64	3.50E−06	2.42E−05
EGR_02699	Cathepsin L1	3051.46	−2.52	0.31	−8.10	5.45E−16	2.37E−14

FDR  < 0.05.

### qRT-PCR results validate RNA-Seq

**Figure 6:**
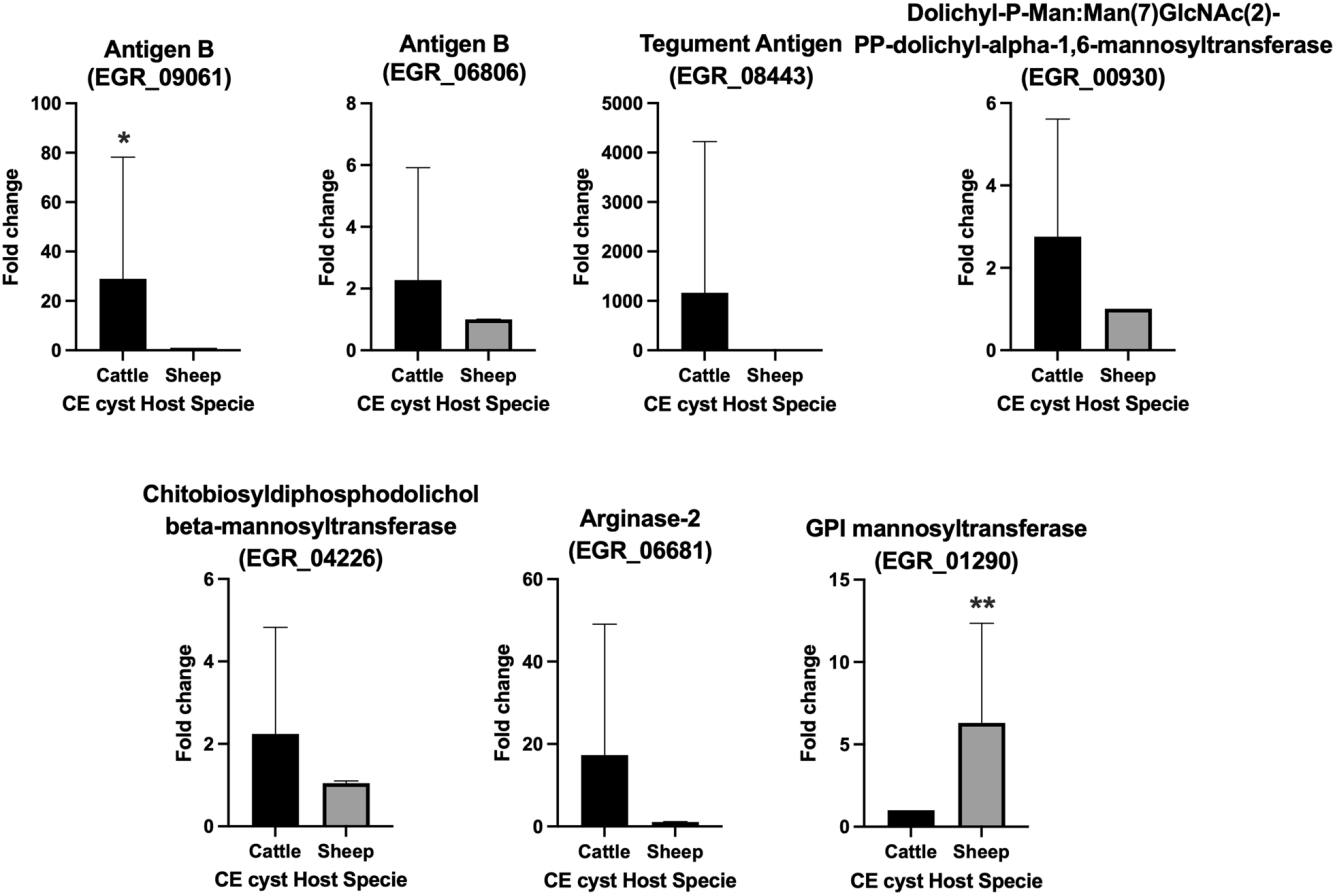
**Fold-change expression of immune related genes in protoscoleces from cattle and sheep liver CE cysts**. qRT-PCR was performed for each gene to validate the RNA-Seq results. Genes were selected from the DE results from the comparison of PSC from cattle v/s sheep liver CE cysts, with FDR 0.05 and without considering log_2_FoldChange cut-off. The housekeeping gene TBP was used to normalize relative expression. Statistically significant differences were determined by Mann–Whitney test. *P  < 0.05, **P  < 0.01. CE  =  cystic echinococcosis, PSC  =  protoscoleces.

For the validation of our results from the differential expression of immune modulation related genes, seven genes were selected from above; antigen B (EGR_09061 and EGR_06806), tegument antigen (EGR_08443), dolichyl-P-Man:Man(7)GlcNAc(2)-PP-dolichyl-alpha-1,6-mannosyltransferase (EGR_00930), chitobiosyldiphosphodolichol beta-mannosyltransferase (EGR_04226), arginase-2 (EGR_06681), GPI mannosyltransferase (EGR_01290) and one housekeeping gene, TBP (EGR_02554). The criteria for choosing these 7 genes were at least 1.5 log_2_FoldChange and FDR  < 0.05 in the DE results. All selected genes were overexpressed in one host, with statistically significant differences for the antigen B (EGR_09061) and GPI mannosyltransferase (EGR_01290) genes. The results from the RNA-Seq confirmed our qRT-PCR results (Figure 6).

## Discussion

In the present study, we employed RNA-Seq Illumina technology to uncover transcriptomic changes in *E. granulosus* s.s. protoscoleces obtained from sheep liver and lung CE cyst samples, comparing differential expression with PSC obtained from cattle liver PSC.

The haplotype network obtained is similar to other *E. granulosus* s.s. networks published, with haplotypes EgAus03, EgRUS7 and EgCL22 already reported [7, 29], whereas EgCL34 is a new haplotype not described before this study. There were no differentially expressed genes particular to any cox1 haplotype.

As we stated before, fertile sheep CE cysts are relatively easy to find, because the fertility rates are higher than in cattle. In our work, after the examination of nearly 1000 cattle CE cysts, only few CE cysts were fertile and their extracted RNA quality was low. In the SRA file database, we only found 4 samples belonging to *E. granulosus* s.s. PSC from cattle. From that pool of samples, 1 of them was equal to our samples, 3 of them were subjected to different conditions. It is important to note that differences in gene expression may occur among the various stages of strobilation. It is reported that there are differential expressions in proteins (genes not analyzed) through the strobilation. These proteins are mainly involved in metabolic, regulatory, and signaling processes, and not in immune modulation (for instance antigen B or tegumentary antigen) [30]. The cattle PSC data that was used in this work, was previously found to have differentially expressed genes associated with molecular functions of signal transduction, enzymes, and protein modifications. A comparison of our findings related to immune modulation genes found that not one of these genes were differentially expressed with a fold change over 1.5 [19].

Since CE cysts show different fertility rates according to the mammalian host [4, 5], we chose to focus on immune modulation related genes. Regarding these immune modulation related genes, we found them upregulated in PSC from cattle liver CE cysts when compared to sheep liver CE cysts. Two antigen B related genes (EGR_09061 and EGR_06806) and a tegumentary antigen (EGR_08443) gene transcription were upregulated in PSC from cattle liver CE cysts. It is known that antigen B and tegumentary antigen play a key role in the evasion of the host immune response. For example, antigen B induces the synthesis of specific IgE and IgG4, and inhibits polymorphonuclear cell (PMC) chemotaxis [31]. Moreover, antigen B-stimulated PMC produced IL-4 and IL-13 but did not produce IL-12 [32]. The tegumentary Antigen is also involved in the evasion of immune response by inhibiting chemotaxis, inducing preferentially IL-4 producing lymphocytes, and non-complement fixing antibodies like IgG4 [33]. Genes encoding both proteins were already found expressed in the transcriptome of PSC from sheep liver CE cysts [16]; to our best knowledge this was not previously described for PSC from cattle CE cysts.

Arginase-2 (EGR_06681) was also upregulated in PSC from cattle liver CE cysts. It is described that arginase-2 contributes to the immune modulation of *H. pylori* by limiting macrophage iNOS protein expression, limiting NO production, and mediating macrophage apoptosis [34]. Also, arginase-1 and arginase-2 isoform expressions were measured in multiple myeloid cells from the peritoneum of mice infected by *E. granulosus*. No differences in the expression of arginase-2 was found. However, arginase-1 isoform was differentially expressed, promoting immune modulation of *E. granulosus* in mice by inhibiting the expression of T cell receptor CD3ζ chain and antagonism against iNOS [35]. The expression of arginase was not measured in PSC and to the best of our knowledge, no previous work investigated the expression of arginase-2 in PSC. In our work, the arginase-2 gene (but not arginase-1) was upregulated in PSC from cattle CE cysts compared to PSC from sheep CE cysts. It would be interesting if arginase expression is also upregulated in the adventitial layer of CE cyst or in the surrounding parenchyma.

Three genes related to some mannosyltransferases were also upregulated, namely those coding for dolichyl-P-Man:Man(7)GlcNAc(2)-PP-dolichyl-alpha-1,6-mannosyltransferase (EGR_00930), Dolichol-phosphate mannosyltransferase (EGR_01475) and chitobiosyldiphosphodolichol beta-mannosyltransferase (EGR_04226). However, none of these proteins have been associated with immune response evasion, although it is known that Mannose binding lectin plays a key role in the innate immune response, this lectin binds to surface carbohydrate then activates the lectin complement pathway [36]. As observed in other organisms, mannoses have already been described in *E. granulosus* s.l. [37]. Interestingly, the method used to detect mannoses-ag was lectin blotting. Our finding suggests that overregulation of mannosyltransferases is a mechanism to modify mannoses to evade the lectin pathway and is an important component of the immune response evasion.

Immune modulation related genes upregulated in PSC from sheep liver CE cysts compared to PSC from cattle liver CE cysts were also found. A basigin gene (EGR_08038) was found to be upregulated in PSC from sheep CE cysts. Basigin (also called EMMPRIN) is a transmembrane glycoprotein that belongs to the immunoglobulin superfamily and its carbohydrate portion is recognized by lectins such as galectin-3 and E-selectin [38]. Knockout mice for basigin have more active lymphocyte reactions, compared to the wild type mice, suggesting that suppression of the lymphocyte response [39] is because of the inhibitory effect of basigin over the Nuclear factor of activated T-cells (NFAT) [40]. The detection of basigin in *E. granulosus* PSC was previously done by mass spectrometry using CE cyst from mice infected with PSC of cattle CE cysts from the lungs and liver; no PSC from sheep CE cyst were included in that work. In *F. hepatica* an RNA-Seq experiment identified a transcript that shares sequence similarity with the basigin gene suggesting this transcript that co-evolved with basigin could be important in the regulation of the host immune response [41]. This mechanism may also be shared by other flatworms like *E. granulosus* s.l.

Another upregulated gene in PSC from sheep CE cysts is cathepsin L1. In *F. hepatica* is the major component found within the ES products from adults, showing suppression of pro-inflammatory cytokines; it is used as a vaccine antigen that reduces fluke burdens in cattle by 48.2% [42]. This cathepsin peptidase shows the highest degradation of purified fibrinogen among other cathepsins in *F. hepatica* [43]. Also, in *Echinostoma caproni*, a cathepsin L-like peptidase is involved in the degradation of surface-trapped antibodies of this parasite. Cathepsin L1 has not been characterized before in *E granulosus*, but a similar protease, Cathepsin B, has been cloned and expressed [44].

We previously reported that both cattle and sheep with multiple CE cysts in the liver and lungs can either harbor the same *E. granulosus* s.s. *cox1* haplotype or as many as 5 different *cox1* haplotypes [7]. Principal component analysis shows that PSC obtained from liver and lung CE cysts, either of the same *cox1* haplotype (for example, EgCL22) or different *cox1* haplotypes do not group in any particular way, indicating that cox1 haplotypes or viscera in which the parasite is hosted, have no influence on their gene expression. Conversely, there is a striking differential gene expression between PSC found in sheep and cattle liver CE cysts, indicating that the microenvironment from the intermediate host has an influence on parasite gene expression including genes related to the evasion of the immune response. This is particularly relevant, as *E. granulosus* s.s. CE cysts found in sheep usually are fertile whereas cattle CE cysts rarely are [10]. One of the limitations of our study, is that *Echinococcus granulosus* s.s. has high intraspecific variability [29], which could affect our comparisons between our field samples and the data obtained from the SRA. Since this work was done with naturally infected livestock, variability between samples will always be an issue; however, the data we obtained from the liver and lung cysts in the same infected animal minimizes this effect, as there were no major differences in gene expression between PSC from the same haplotype and affected organ.

It is plausible that the differential gene expression in immune modulation genes is one of the factors involved in the low survival of PSC in the cattle host, contributing to the higher prevalence of non-fertile CE cysts compared to sheep CE cysts.

We concluded that *E. granulosus* s.s. PSC obtained from the liver and lungs do not show differentially expressed genes between them. Instead, we found that the host species is the main factor in the differential expression of immune-related genes. We suggest that the success of the parasite survival in the intermediate host depends on the particular response to the host immune system. The apparently different immune modulation mechanisms between sheep and cattle PSC should be considered when control strategies are planned.

## Supplementary Information

**Additional file 1: Sequencing summary of samples Li01–06 and Lu01–06. Total bases, read counts and Phred scores are shown.**

**Additional file 2: Mapping % summary of samples Li01–06 and Lu01–06 to the reference genome (GCA_000524195.1 ASM52419v1).**

**Additional file 3: Differentially expressed genes in protoscoleces from liver and lung sheep CE cysts (comparison between Li01–06 v/s Lu01–06 samples).**

**Additional file 4: Differentially expressed genes in protoscoleces from cattle and sheep liver CE cysts (comparison between B1–4 v/s Li01–06 samples).**

**Additional file 5: GO terms in protoscoleces from cattle and sheep liver CE cysts (comparison between B1–4 v/s Li01–06 samples) in genes with FDR 0.05 and without considering log**_**2**_**FoldChange cut-off.**

**Additional file 6: Immune modulation genes evaluated in protoscoleces samples obtained from liver and lung CE cysts.**

**Additional file 7: Primers used for selected genes in qRT-PCR validation assays.**

## Data Availability

The dataset supporting the conclusions of this article is available in GenBank under the Accession Number PRJNA736768.

## Abbreviations

CE: cystic echinococcosis
PSC: protoscolex
ES: excretory-secretory
DEA: differential expression analysis
GO: gene ontology
SRA: short read archive
PCA: principal component analysis
DE: differentially expressed
s.l.: Sensu lato
s.s.: Sensu stricto
